# Effects of Dietary Supplementations of Vitamin C, Organic Selenium, Betaine, and Pomegranate Peel on Alleviating the Effect of Heat Stress on Growing Rabbits

**DOI:** 10.3390/ani14060950

**Published:** 2024-03-19

**Authors:** Salma H. Abu Hafsa, Gerardo Centoducati, Ayman A. Hassan, Aristide Maggiolino, Mona M. M. Y. Elghandour, Abdelfattah Z. M. Salem

**Affiliations:** 1 Livestock Research Department, Arid Lands Cultivation Research Institute, City of Scientific Research and Technological Applications, New Borg El-Arab, Alexandria 21934, Egypt; hashim_salma@yahoo.com; 2 Department of Veterinary Medicine, University of Bari, S.P. per Casamassima km 3, 70010 Valenzano, Italy; aristide.maggiolino@uniba.it; 3 Agricultural Research Center, Department of By-Products Utilization Research, Animal Production Research Institute, Giza 12619, Egypt; aymanan19@hotmail.com; 4 Facultad de Medicina Veterinaria y Zootecnia, Universidad Autónoma del Estado de México, Toluca 50000, Mexico; mmohamede@uaemex.mx

**Keywords:** rabbits, vitamin C, selenium, betaine, pomegranate peel, heat stress, cecal fermentation

## Abstract

**Simple Summary:**

Heat stress has become a global concern, one of the major environmental stressors, and causes substantial economic loss in the modern rabbit industry. Heat stress is caused by several factors such as high environmental temperature and humidity, causing a series of unfavorable changes in nutritional, physiological, and immunological functions, blood biochemical indexes, and antioxidant capacity. These factors negatively affect the productive performance and cecal fermentation and microbiota in rabbits. Thus, this study aimed to assess the potential use of feed additive strategies (e.g., vitamin C, organic selenium, betaine, and pomegranate peel) to mitigate heat stress on rabbits, and to evaluate rabbit performance, antioxidant status, and cecal fermentation and microbiota under heat stress. Rabbits fed diets supplemented with vitamin C, organic selenium, betaine, and pomegranate peel demonstrated significantly improved growth performance, nutrient digestion, concentrations of total and individual volatile fatty acids, and total cecal beneficial bacterial count. In contrast, there was a significant decrease in NH3-N concentration and pathogens’ counts (e.g., *Enterococcus*, *coliforms*, and *E. coli*) in the rabbit cecum. Blood biochemistry parameters and antioxidant capacity of rabbits supplemented with vitamin C, organic selenium, betaine, and pomegranate peel were observed to be improved in comparison to those in the control group. The results showed the significant impact that vitamin C, organic selenium, betaine, and pomegranate peel supplements play in alleviating heat stress and enhancing the positive effects on cecal fermentation and microbiota, antioxidant status, and rabbit performance. However, betaine supplementation had a superior positive impact on the nutritional and physiological profile of heat-stressed rabbits.

**Abstract:**

The aim of this study was to investigate the biological activity and sustainable biorefinery development of vitamin C, organic selenium, betaine, and pomegranate peel on the performance, cecal fermentation, and antioxidant status of growing rabbits to alleviate the negative impacts of heat stress. A total of 105 male rabbits at 35 days old with an average weight of 752.55 ± 27.56 g were randomly assigned to five groups (21 rabbits in each). The experimental dietary groups included a control group fed a basal diet without additives (control group) and four treated groups, A, B, C, and D, fed a basal diet supplemented with either a 1000 mg vitamin C/kg diet, a 25 mg organic selenium/kg diet, a 1000 mg betaine/kg diet, or a 20 g pomegranate peel/kg diet, respectively. No negative group (not heat-stressed) was included in the trial. Rabbits given A, B, C, and D supplements showed a significant increase (*p* < 0.05) in growth performance, nutrient digestion, concentration of total volatile fatty acids (VFA), acetic, and propionic acids, and total bacterial count, and a significant decrease (*p* < 0.05) in NH_3_-N concentration, *Enterococcus*, *coliforms*, and *E. coli* counts in the cecum of rabbits. Total protein, albumin, globulin, superoxide dismutase (SOD), catalase (CAT), reduced glutathione (GSH), and glutathione peroxidase (GPx) were significantly (*p* < 0.05) higher in groups supplemented with A, B, C, and D supplements compared to those in the control group. Total cholesterol, triglycerides, creatinine, urea, and malondialdehyde (MDA) were significantly (*p* < 0.05) lower in groups supplemented with A, B, C, and D supplements compared to those in the control group. In conclusion, the finding showed that the supplementation of vitamin C, organic selenium, betaine, and pomegranate peel played a significant role in alleviating heat stress and had a further beneficial impact on rabbit performance, cecal fermentation and microbiota, and antioxidant status. However, betaine supplementation had a superior positive effect on the nutritional and physiological profile of heat-stressed rabbits.

## 1. Introduction

Recently, climate change has posed a severe threat to livestock productivity, in particular due the predicted global rise in ambient temperature and the magnitude of an animal response to increasing physiological temperature [1]. In Egypt, high temperatures ranging between 25 and 45 °C in summer are a major limiting factor that negatively affects rabbit production performance. Heat stress is a condition in which rabbits are unable to maintain a balance between heat production and emission. A high ambient temperature in summer can easily cause heat stress in rabbits, resulting in a series of negative impacts on rabbit productivity [2]. The interaction of various factors, including high temperature, humidity, radiant heat, and air velocity, leads to heat stress. Heat stress is mostly caused by high ambient temperature among these factors [3]. Rabbit body temperature under normal condition is between 38.5 and 39.5 °C, with individual variations ranging from 0.5 to 1.2 °C. The optimal temperature range for rabbits is between 15 and 25 °C, while the optimum humidity is between 55 and 65%; heat stress occurs at temperatures over 30 °C, and when the temperature is higher than 35 °C, rabbits are unable to regulate their body temperature, ultimately leading to heat failure [4]. As is known, heat stress has multiple detrimental effects on rabbit health and productive performance, and it has been reported that heat stress induces a decrease in daily weight gain (20–25% less), a decrease in feed conversion ratio (up to 15%), and an increase in mortality rate. Moreover, it causes a reduction in reproductive performance, negatively affects carcass characteristics and meat quality, and leads to changes in blood metabolites and enzymatic and hormonal secretions [5,6]. Thus, heat stress causes a great challenge for the rabbit industry, especially with global warming [1]. Heat-stressed animals are required to be provided with vitamin resources to correct the protein and energy’s negative balances during the hot weather season in Egypt [7]. Nutritional approaches may support animals in maintaining homeostasis or avoiding nutrient deficiencies resulting from heat stress [8]. Of these, vitamin C, organic selenium, betaine, and pomegranate peel as anti-heat stress feed additives have been documented and proven to be effective mitigation strategies for heat stress in rabbits [9]. Vitamin C and selenium are well known for their ability to scavenge free radicals, hence reducing oxidative stress [8]. It is widely known that vitamin C supplementation provides antistress and antioxidant properties [10] and can mitigate heat stress and increase the growth performance in rabbits [11]. Vitamin C participates in the redox reaction and scavenges the free radicals generated by peroxidation in vivo to prevent tissue cells from oxidative damage; in the diet, it prevents the body temperature increasing in heat-stressed rabbits, increases T3 and T4 concentrations, and decreases cortisol glucose, corticosterone, and MDA levels in blood, thereby reducing the detrimental impact of heat stress in vivo [12,13]. Selenium is a vital trace element for mammals and is involved in numerous important physiological processes, including immunity, growth, and reproduction [14]. Selenium is an important component of multiple selenium proteins, including glutathione peroxidase (GSH-Px) and thioredoxin reductases (TrxRs) [15,16], and GSH-Px is a major phase II detoxification enzyme that can reduce the level of lipid peroxidation. Thus, an adequate amount of selenium in the diet of rabbits is essential for the antioxidant and immune function of rabbits [17] and for their neuropsychological functions [18]. A previous study [19] reported that giving heat-stressed rabbits a diet containing 0.034 mg/kg of organic selenium reduced their rectal temperature by about 0.5 °C. On the other hand, serum protein, albumin, and GSH-Px activity increased, while MDA significantly decreased. Betaine (trimethylglycine, BET) is a well-known methyl donor engaged in numerous important biochemical pathways, which provides BET with many of its physiological activities [20,21]. It is known to be the most effective osmoprotectant, acting as an osmolyte, and is able to confer cell protection against environmental stresses [22,23]. In fact, high temperatures may lead to dehydration due to water imbalance and osmotic changes in animals’ body cells. Due to its regulation of osmosis, betaine may help maintain cell integrity and hydration under thermal stress [24]. Betaine is a natural anti-heat stress agent in the animal and poultry industries. Pomegranate by-products (*Punica granatum* L.) can be a reliable supply of nutrients and antioxidants for feeding animals such as rabbits [25]. Pomegranate and its derivative parts have already been shown to have several phenolic activities [26]. Pomegranate peel (PP) has attracted attention for its pronounced antioxidant and antibacterial properties due to its high concentration of bioactive compounds, which has made it associated with many health benefits [27]. Tannins and flavonoids, two bioactive substances that are prevalent in the polyphenol class of antioxidants, are included in PP. It has been proposed that antioxidant activity plays a crucial role in a number of pharmacological activities, such as those that fight inflammation, atherosclerosis, and aging. Antioxidant supplements are becoming a popular treatment strategy in reducing the risk of disease due to their ability to prevent free radical damage. Therefore, nutritional strategies have the potential to be used for alleviating heat stress, with the advantages of safety, high efficacy, and few toxic side effects. Thus, the main objective of this study was to determine possible strategies for mitigating heat stress using feed additives such as vitamin C, organic selenium, betaine, and pomegranate peel and to evaluate rabbits’ performance, antioxidant status, and cecal fermentation and microbiota under heat stress.

## 2. Materials and Methods

### 2.1. Animals and Experimental Groups

The present study was performed at Nuobaria Station, Animal Production Research Institute (APRI), and all the experimental procedures that used the rabbits for this trial were approved by the Institutional Animal Care and Use Committees of City Scientific Research and Technological Applications (Protocol No. 57-2Z-3022), Alexandria, Egypt. One hundred and five weaned New Zealand White (NZW) male rabbits (752.55 ± 27.56 g, 35 days old) were used in this experiment. The growing rabbits were randomly divided into five treatment groups (n = 21 each) with seven replicates (n = 3 each replicate). Experimental dietary groups included a control group fed a basal diet without additives (control group), and four treated groups, A, B, C, and D, fed a basal diet supplemented with either a 1000 mg vitamin C/kg diet (Microvit^®^ C Promix 1000, Adisseo, Paris, France), a 25 mg organic selenium/kg diet (Sel-Plex^®^, containing seleno-methionine as the main seleno-compound, Alltech Inc., Nicholasville, KY, USA), a 1000 mg betaine/kg diet (Betafine^®^, Adisseo, Paris, France) (BET group), or a 20 g pomegranate peel/kg diet, respectively. In order to prepare pomegranate peels for use as a feed additive in this investigation, fresh pomegranate fruits were supplied from the local market (Alexandria, Egypt). After manually peeling, peels were separated, rinsed with tap water and air-dried for 5 days. Then, dried peels were pulverized with a grinder. After passing through a sieve, the powder was put into a glass bottle covered with aluminum foil and stored at room temperature until use. Each additive was first combined with 1 kg of diet and then gradually mixed with the remaining diet to reach a homogeneous inclusion level. The experiment lasted for 70 days during the summer season, with temperatures ranging from 29.8 °C to 32.9 °C and humidity ranging from 75.5 to 81.4%. The basal diet was formulated to cover the recommended nutrient requirements of rabbits according to NRC [28]. The composition of the experimental diet is shown in Table 1. The diet ingredients did not contain antibiotics. All rabbits used in this experiment received vaccinations against common rabbit diseases in accordance with the traditional program used for rabbits before the start of the experiment. Throughout the experiment, the rabbits were housed individually in galvanized wire cages (L 35 cm × W 35 cm × H 60 cm) equipped with conventional feeders and an automatic system of nipple drinkers. Environmental conditions (temperature, management, and hygiene) were all the same for all rabbits. Each morning, feces and urine were removed from the floor of the rabbit tray. 

### 2.2. Temperature–Humidity Index Calculation

The severity of heat stress in rabbits was detected using the following method. An automatic thermohygrometer (OF 14:140, H 10–99%; TFA Dostmann GmbH and Co. KG, Wertheim, Germany) was installed at a height 30–50 cm above the rabbit cages and was used to measure ambient temperature (AT) and relative humidity (RH) at a constant time every day during the study period. The AT and RH were recorded every day at 14.00 in order to calculate the temperature–humidity index (THI) values, which represent the level of heat stress experienced by rabbits throughout the experiment period. The subsequent equation was employed to detect the THI values [29]: (1.8 × AT + 32) − (0.55 − 0.55 × RH) × [(1.8 × AT + 32) − 58], where AT is the environmental temperature expressed in degrees Celsius, so that the term (1.8 × AT + 32) represents the conversion of temperature data in degrees Fahrenheit, and RH is the relative humidity as a fraction of unit [30]. The attained THI values were then classified as follows: <82 (absence of heat stress), 82 ≤ 84 (moderate heat stress), 84 ≤ 86 (severe heat stress), and 86 and more (very severe heat stress). These values followed calculations recorded by Marai and Rashwan [31] in small animals (rabbits and poultry).

### 2.3. Growth Performance

Individual weights of rabbits were recorded weekly to determine the final body weight (FBW), and feed intake (FI) was recorded daily. Average daily gain (ADG) and feed conversion ratio (FCR; g feed/g gain) were calculated [32]. The mortality rate was recorded daily, and the percentage was recorded for each group at the end of the experiment.

### 2.4. Digestibility Trial

In the last week of the experiment, a digestibility trial was conducted to determine the apparent nutrients’ digestibility according to Perez et al. [33]. Seven rabbits per treatment group were randomly selected and kept separately in metabolic cages for a 5-day digestibility trail. The quantity of feed offered and the amount remaining were recorded daily and used to determine daily feed intake. After that, feces collected daily before feeding in the morning for the five consecutive days were weighed, thoroughly mixed, and stored at −20 °C. Representative samples of daily fecal matter collected were oven-dried at 70 °C for 48 h, then ground through a 1 mm mill screen opening. Laboratory analyses were carried out on feed and fecal samples using the standard AOAC procedures to determine organic matter (OM), dry matter (DM), crude protein (CP), and crude fiber (CF) content [34]. For neutral detergent fiber (NDF) and acid detergent fiber (ADF), a sequential method was determined with the ANKOM fiber analyzer as described by Maggiolino et al. [35] and according to Van Soest et al. [36]. The nitrogen-free extract (NFE) value was calculated by a different method. The digestible energy (DE) was estimated according to the equation of Noblet [37] as follows: DE (kcal/kg diet) = 5.28 DCP (g) + 9.51 DEE + 4.2 (DCF + DNFE).

Where, DCP = digestible crude protein, DEE = digestible ether extract, DCF = digestible crude fiber, and DNFE = digestible nitrogen free extract.

### 2.5. Carcass Characteristics 

Seven rabbits from each group were randomly chosen and starved for 12 h (only ad libitum water), then weighted and slaughtered. The Islamic method was followed for slaughtering the rabbits [38]. Following complete bleeding, the viscera, tail, and pelt were removed, and the carcass and all of its parts were then weighed as total edible parts. The edible giblets (heart, liver, spleen, lung, and kidneys) were measured as a percentage of live body weight. The dressing percentage was calculated by dividing the hot-dressed carcass weight by the preslaughter weight and expressed as a percentage. Moisture content was determined in meat samples in an oven at 70 °C to a constant weight [39]. The CP content (N 6.25) was determined according to Kjeldahl’s method (AOAC, 2005; method 978.04) [40]. Ether extract was determined according to the Soxhlet extract method using petroleum ether as an extract agent (40–60 °C) (AOAC, 2005; method 930.09) [41]. Total ash content was assayed by incinerating the samples in a muffle furnace at 550 °C (AOAC, 2005; method No. 930.05) [42].

### 2.6. Cecal Microbiota and Fermentation Patterns

Seven rabbits were taken from each group for the measurement of cecal weight and length, cecal fermentation, and bacterial count. After the rabbits were euthanized, the cecum was carefully excised and emptied with a gentle pressure in order to remove any digesta remaining in the cecum to measure its weight and length. The full and empty weights of the cecum (*g*/*g* body weight) and its length (cm) were calculated. Immediately, the cecal content was strained through two layers of sterile gauze and the resultant strained liquor was used to determine the pH levels by an electronic digital pH meter (GLP 21 model; CRISON, Barcelona, Spain). Following this, the mixture was centrifuged at 7000× *g* for 10 min at 20 °C. The supernatant fluid was divided into two portions. One part was treated with a solution of 5% orthophosphoric acid (*v*/*v*) plus 1% mercuric chloride (*w*/*v*) (0.1 mL/mL sample) to measure total VFA concentration and individual VFA proportions, while the other was acidified with 0.2 M hydrochloric acid solution (1 mL/mL sample) to be used to measure the ammonia nitrogen (NH_3_-N) concentration. According to Eadie et al. [43], total VFA concentration was determined by steam distillation. According to the method described by Mathew et al. [44], the concentration of VFA was analyzed using high-performance liquid chromatography (HPLC; Model Water 600; UV detector, Millipore Crop., Burlington, MA, USA). The concentrations (mmol/L) of acetic, propionic, and butyric acids were calculated once the results of the concentration of the particular VFA had been received. The cecal NH_3_-N was determined by using spectrophotometry, as described by Chaney and Marbach [45]. Ceca were quickly removed, and their contents were collected in sterile tubes. Tenfold dilutions of each sample were serially prepared in phosphate-buffer solution and poured directly onto Petri dishes containing culture media. Total bacteria and *Enterococcus* sp. were cultured in de Man Rogosa Sharpe (MRS) agar and incubated under anaerobic conditions for 48 h at 37 °C. Bacteria were plated on MacConkey agar and incubated aerobically for 24 h at 37 °C. Bacterial colony-forming units (CFU) in the Petri dishes were counted using a range of 30–300 cfu/g, depending on the growth characteristics of the bacterial species. The counts were expressed as log cfu/g.

### 2.7. Blood Sampling, Biochemistry, and Antioxidant Status

At the end of the study, samples of blood were obtained from the seven slaughtered rabbits and placed in nonheparinized sterile tubes. The samples were centrifuged at 2000× *g* for 15 min after being allowed to coagulate at room temperature for 30 min. Serum was separated and stored at −20 °C until analysis. Total protein, albumin, globulin, cholesterol, triglycerides, creatinine, and urea were determined [46], while globulin was calculated as the difference between total protein and albumin [47]. 

Superoxide dismutase (EC 1.15.1.1) activity was determined as described by Dinardo et al. [48]. The enzymatic activity was based on the 50% inhibition rate of epinephrine autooxidation at 480 nm. The stimulation of epinephrine autoxidation by traces of heavy metals present as contaminants in the reagents or by the other metals under investigation was prevented by adding 10^−4^ *M* EDTA in the buffer to chelate those ions. The activity of SOD was expressed as units per milliliter (U/mL) of protein. The measurement of H_2_O_2_ disappearance was used to determine the presence of catalase (EC 1.11.1.6) as described by Aebi [45]. For this, 50 µL of diluted enzyme extract and 1.5 mL of 0.1 M phosphate buffer (pH 7) were added to a 3 mL reaction mixture along with 0.5 mL of 75 mM H_2_O_2_. The amount of H_2_O_2_ that was decomposed allowed for the computation of enzyme activity after a one-minute observation of a reduction in absorbance at 240 nm. Glutathione peroxidase (GPx, EC 1.11.1.9) activity was measured as reported by Maggiolino et al. [49]. The reaction measured the rate of reduced glutathione oxidation by tert-butyl hydroperoxide, catalyzed by GPx. Reduced glutathione was maintained at constant concentration by the addition of exogenous glutathione reductase and NADPH, which converted the oxidized glutathione to reduced glutathione. The rate of oxidized glutathione formation was then measured by the change in the absorbance of nicotinamide adenine dinucleotide phosphate reductase (NADPH) at 340 nm. The activity of GPx in plasma was expressed, respectively, as nmol of NADPH oxidized/min per mL and nmol of NADPH oxidized/min/mg of protein. Protein concentration was determined by Bradford’s method using bovine serum albumen (BSA) as a standard [50]. 

To perform the MDA assay in plasma, it was homogenized with a malondialdehyde (MDA) lysis buffer and a thiobarbituric acid (TBA) solution was added to each sample and incubated at 95 °C for 60 min [51]. Then, each reaction mixture was placed into a 96-well plate to measure the absorbance at 532 nm using a Glomax Multi Detection System (Promega, Madison, WI, USA). Results were expressed as nanomoles/dL. Lipid peroxidation in organs was determined by assaying the MDA levels [52]. It was determined by the reaction of MDA with thiobarbituric acid (TBA) to form a colorimetric (532 nm) product, proportional to the MDA present. 

### 2.8. Statistical Analysis

The data set was tested for normal distribution and variance homogeneity (Shapiro–Wilk). Data were statistically analyzed in a randomized complete block design using the general linear model procedures of SAS/STAT (Statistical Analysis System, version 15.1, SAS Institute Inc., Cary, NC, USA) [53]. Statistical significance was considered when the *p*-value was less than 0.05. Data obtained were tested by an analysis of variance with one-way design to test the treatment at each sampling according to the following model:

Y_ijk_ = μ + T_i_ + ε_ijk_

where y_ijk_ denotes the measured value, μ is the overall mean effect, T_i_ is the i^th^ treatment effect, and ε_ij_ denotes the random error associated with the j^th^ rabbits allocated to the i^th^ treatment. Results are presented as least-squares means.

## 3. Results

**Figure 1:**
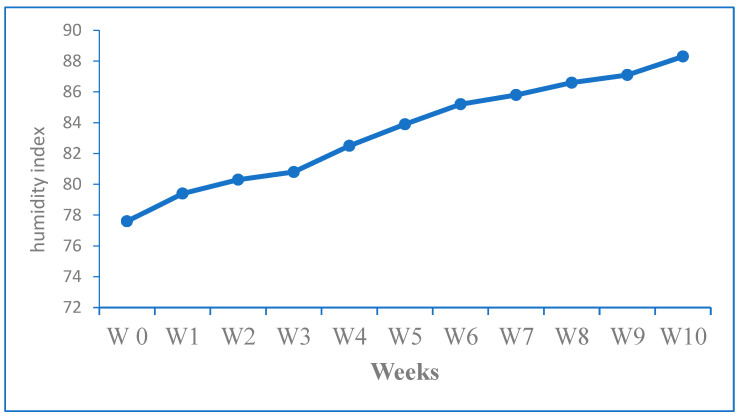
Calculated temperature and humidity index (THI) throughout the experiment period.

The results of the present study estimated that THI values ranged from 85.81 to 88.33 ^◦^F during the last four weeks of the experiment, indicating a severe level of heat stress on the rabbits. Additionally, the overall average THI value was 83.41 °F, indicating severe heat-stress initiation condition (Figure 1). 

The effect of supplementing rabbits’ diet with A, B, C, and D supplements on growth performance is shown in Table 2. 

The diet supplemented with A, B, C, and D supplements significantly improved (*p* < 0.05) final body weight, average daily gain, and FCR but had no effect on feed intake during heat stress compared to the control group, whereas a betaine-supplemented diet (group C) had the largest (*p* < 0.05) final body weight, average daily gain, as well as a better FCR compared to all treatment groups. It was noted that among the different experimental groups, organic selenium- and betaine-supplemented rabbits (group B and C) exhibited the lowest mortality rate.

The digestibility coefficient results of the nutrients are shown in Table 3. The digestibility coefficients of DM, CP, CF, NFE, NDF, and ADF were significantly increased (*p* < 0.05) in supplemented groups with A, B, C, and D supplements, whereas the diet supplemented with betaine (group C) had the highest (*p* < 0.05) values for all nutrient digestibility parameters compared to all treatment groups. However, there were no significant differences in NFE and ADF among the treated groups. 

The control group had a considerable decrease in carcass weight and dressing percentage, but an increase in the relative weight of the liver and kidneys under heat stress (Table 4). Carcass weight and dressing percentage were significantly (*p* < 0.05) increased in response to dietary supplementation with A, B, C, and D supplements compared to the nonsupplemented group. The betaine group (group C) had a higher carcass weight and dressing percentage than the other treatment groups. Due to heat stress, the relative weight of the liver and kidneys increased (*p* < 0.05) in the control group. Relative weights of the liver and kidneys decreased significantly (*p* < 0.05) when supplementing the rabbit’s diet with A, B, C, and D supplements compared with the control group. The composition of the meat carcass for the control group showed a significant (*p* < 0.05) increase in moisture and ether extract contents but a decrease in protein content compared to all supplementation groups under heat stress. Conversely, moisture and ether extract contents decreased significantly (*p* < 0.05) in response to the group supplemented with A, B, C, and D supplements. However, the ash values were not different in any of the treatment groups.

Cecal fermentation patterns and microbiota affected by dietary supplementation of A, B, C, and D supplements are shown in Table 5. The cecal pH and butyric acid values were not different among all treatments during heat stress. During fermentation under the influence of applied rations, the addition of all different experimental supplements of A, B, C, and D supplements to the rabbit rations significantly increased total VFA and acetic and propionic acids in cecal contents (*p* < 0.05), with low values in NH_3_-N (*p* < 0.05). The supplementation of A, B, C, and D supplements to the rabbit rations resulted in a significant (*p* < 0.05) decrease in the counts of *Enterococcus* sp., total coliforms, and *E. coli*, while dramatically (*p* < 0.05) increasing total bacterial count in the cecum of rabbits.

Serum biochemical and antioxidants indices affected by the supplementation of A, B, C, and D supplements are shown in Table 6. Supplementing with A, B, C, and D supplements resulted in a significant (*p* < 0.05) increase in the levels of total protein, albumin, and globulin compared to those in the control group, while the betaine group had the highest levels of total protein, albumin, and globulin compared to the other treatment groups. The betaine group had the lowest (*p* < 0.05) concentration of total cholesterol, triglycerides, creatinine, and urea during heat stress, followed by those in groups A, B, C, and D supplements compared to those in the control group. Serum SOD, CAT, GSH, and GPx were considerably higher in the groups given A, B, C, and D supplements than in the control group. However, the supplementary groups had a lower MDA level than the control group under heat stress.

## 4. Discussion

Egypt’s summer months, especially July and August, offer mild subtropical weather. The mean air temperature values in this study indicated that all rabbit groups were experiencing long-term heat stress, with temperatures ranging from 29.8 °C to 32.9 °C and humidity ranging from 75.5 to 81.4%. Long-term exposure of rabbits to such stress can cause physiological and biological damage [54] due to oxidative stress resulting from excessive ROS production and/or impairment of the antioxidant system [55], leading to adverse effects on the performance of control rabbits. The reduction in thermoregulatory parameters of heat-stressed rabbits caused by vitamin supplementation may also have an ameliorating effect on heat-stressed animals by affecting the prostaglandin output. In rabbits, a combination of selenium and vitamin E was also found to minimize the harmful effects of heat stress [56]. Selenium and vitamins as an antioxidant may provide the most favorable effect on physiological parameters in heat-stressed ewes [57] and in sheep [58].

Dietary supplementation with vitamin C, organic selenium, betaine, and pomegranate peel decreased physiological thermoregulatory parameters of heat-stressed growing rabbits, while improving growth performance. Heat stress is associated with decreased body weight gain, feed intake, and feed efficiency in rabbits, which are highly susceptible to excessive heat [59]. Elevated ambient temperature reduces growth performance, probably due to an excess of ROS that oxidize and damage cellular biological molecules, block some ATPase activities, and ultimately result in a range of intestinal tissues impairments [60]. When rabbits were exposed to heat stress, their body weight gain, feed intake, and feed conversion ratio were negatively affected [61]. Ashour [62] found that T3, which was strongly correlated with feed intake and FCR, was significantly lower in the group exposed to high temperatures. It has been demonstrated that vitamin C enhances growth performance, which may be related to its inhibitory effect on prostaglandins creation and the enzymes responsible for the generation of glucocorticoids and corticosterone, which has a detrimental effect on an animal’s ability to grow under stress [63]. It may explain why heat-stressed rabbits gain more weight after consuming water containing selenium and vitamin E, because these nutrients are involved in the synthesis of vital coenzymes (NAD and FAD), which are in charge of the biological oxidative process that generates necessary ATP for protein, fat, and carbohydrate biosynthesis [64]. Supplementing the rabbit diet with nano-Se under severe heat stress can dramatically improve the growth performance because Se can improve feed utilization by regulating the metabolism of carbohydrates, lipids, and proteins [65]. The beneficial effect of supplementing betaine may be attributed to its ability to support intestinal growth and function; this leads to an increased water-binding capacity of the intestinal cells, as well as enhanced changes in the structure of the gut epithelium and enhanced gut strength [66]. The current findings showed that pomegranate peel supplementation improved final rabbit weight gain and FCR and mitigated the detrimental effects of heat stress on control rabbits’ performance. Pomegranate peel’s high natural antioxidant, antibacterial, and eicosapentaenoic acid content is anticipated to shield the rabbit’s gut from heat-induced epithelial barrier damage to tight junctions and permeability dysfunction [67] and pathogens, as well as promote the growth of beneficial intestinal bacteria [68,69]. However, the lack of a negative group in this trial, that is, not heat-stressed, should be considered.

Supplementation with vitamin C, organic selenium, betaine, and pomegranate peel to heat-stressed rabbits positively affected the digestion of nutrients. El-Moniem et al. [70] showed that nutrients’ digestibility was improved in rabbits fed diets supplemented with a 1000 mg betaine/kg diet. Since nutrient absorption processes depend on an intact gut epithelium, an increase in intestinal integrity [71], the capacity of intestinal mucosal cells to retain water [72], and osmotic properties for intestinal cells may all be contributing factors to improved nutrient digestion after betaine supplementation [73]. Furthermore, improved absorption capacity of the intestinal epithelium and increased fermentation activity of intestinal microflora could be responsible for the increase in nutritional digestibility [74]. Generally, among the tested additives, only betaine evoked a significant improvement in nutrient digestibility values. It was suggested that betaine’s beneficial effects could be attributed to its potency in stabilizing cell membranes through interactions with membrane phospholipids, as well as its ability to lower fecal water content and accordingly increase the digestibility of several nutrients [75]. Furthermore, betaine has been demonstrated to improve gut health and function as well as promote intestinal immunity [76]. The positive effect of 0.5% of betaine supplementation on protein digestibility in Nile tilapia could be explained by the increase in nutrient absorptive surface (increase villi height) and increased digestive enzymes [77]. As evidenced by the elevation of rumen total VFA level with betaine administration, an increase in apparent digestibility of DM, OM, CP, NDF, and ADF was related to an accelerated ruminal nutrient degradation. Additionally, supplementing with betaine may facilitate postrumen nutritional absorption [77]. Betaine’s osmoprotective properties allow it to improve the structure and function of the digestive system, as well as increase intestinal cell activity, which aids in the release of digestive enzymes and nutrient digestion [78]. 

Some carcass traits and meat compositions have been observed to be affected by betaine. Betaine has been observed to accumulate in the muscles of pigs [79], thus potentially affecting meat quality. Virtanen [80] reported that the fat percentage in the body was reduced as the amount of betaine in the diet increased due to betaine being implicated in lipid metabolism and possibly interfering with lipid metabolism. Moreover, that author reported that betaine was efficient in increasing breast meat yield. Recently, Chen et al. [81] reported that dietary betaine supplementation to quail improved carcass characteristics, resulting in more lean meat and lower carcass fat content. By enhancing methylation metabolism and stimulating β-oxidation of long-chain fatty acids in the inner mitochondria membrane of muscle cells, betaine can improve the synthesis of carnitine [82].

According to Park and Kim [83], betaine lowered broilers’ excreta’s ammonia gas emission. Nevertheless, excreta gas emissions were connected to the absorption of nutrients, given that the present investigation showed that betaine enhanced nitrogen digestion. Therefore, supplementation with betaine minimized odors in broiler housing due to reduced excreta ammonia gas emission in growing broilers [83]. The favorable effect of betaine supplementation on the activity of carboxymethyl cellulase, cellobiase, xylanase, and pectinase and *F. succinogenes* as dominant fibrolytic bacteria were responsible for the increase in ruminal total betaine content and acetate/propionate ratio [84]. Betaine was shown to be an effective osmolyte in bacteria [85], and its addition to ruminal microbial growth could provide accessible N and methyl groups [86]. Therefore, the administration of betaine yielded a favorable response of the ruminal microbial population, and apparent total-tract nutrient digestibility was observed. Other studies reported that dietary betaine supplementation improved the abundance of intestinal Gram-positive bacteria and the ability of piglets to digest fiber [73], as well as the concentration of total VFA and acetate in the rumen of dairy cows [87]. The higher protease activity and total protozoa and Rb. amylophilus populations were not in agreement with the decreased NH_3_-N concentration, suggesting that additional NH3-N might be utilized to synthesize microbial protein. Furthermore, by supplementing with betaine, the rumen’s total VFA concentration and bacteria population may increase, allowing for improved microbial protein synthesis [88]. Meanwhile, researchers found that supplementing dairy cow feed with betaine (15 g/d) enhanced total VFA and acetic acid proportion while decreasing propionic acid proportion; hence, betaine may be metabolized and converted to acetate in the rumen and enhance the concentration of acetate [77,89]. 

Serum total protein is the sum of albumin and globulin, which are both used to measure the total quantity of protein in the blood. Some components of blood plasma can be utilized as indicators of the health and functional efficacy of some organs, as well as animal immunological efficiency. For instance, total protein has a positive relationship with tissue synthesis in broiler chicks [90]. Enhancing methylation metabolism and promoting- β-oxidation of long-chain fatty acids in the inner mitochondria membrane of muscle cells are two ways that can improve the synthesis of carnitine [82]. Betaine supplementation reduced total cholesterol and triglycerides in ducks [91]. Supplementing commercial ducks’ diet with betaine at 0.5, 1.0, and 1.5 g/kg resulted in lower total cholesterol and LDL levels, as well as an increase in HDL levels [92]. During exposure to heat stress, ample oxygen-derived free radicals are generated, resulting in oxidative damage of macromolecules [93]. The dietary supplementation with vitamin C, organic selenium, betaine, and pomegranate peel increased total protein and albumin concentrations but decreased total cholesterol, triglycerides, creatinine, and urea concentrations in heat-stressed growing rabbits. The decrease in thermoregulatory parameters of heat-stressed rabbits could be attributable to an increase in protein anabolism and a decrease in protein catabolism. Furthermore, the rise in blood metabolites could be attributed to additives that improved overall animal health and feed utilization. Conversely, SOD, CAT, GSH, and GPx activities were significantly increased in vitamin C-, organic selenium-, betaine-, and pomegranate peel-supplemented rabbits compared with those in control rabbits. In contrast, MDA level was significantly lower in all tested additives compared to the control. Due to its soluble nature, Vitamin C actively participates in the structure of organic compounds because it is situated at the membrane level, reducing oxidative damage, and the peroxidation of fatty acids and phospholipid components [63]. One of the major factors causing animal inflammation and immunological dysfunction is oxidative stress. The potential of dietary Se to improve animal antioxidant status is directly tied to its ability to moderate the inflammatory response generated by heat stress. The major cause of oxidative stress is an imbalance between the body’s generation of oxides and its antioxidant defense system [94]. Betaine’s capacity to prevent mitochondrial lipid peroxidation, scavenge free radicals, and preserve optimal cellular functions is what account for its antioxidant action [95]. Lower rectal temperatures and respiration rates were seen after the addition of betaine hydrochloride (2 g/kg feed), which is suggestive of poultry raised in various environmental circumstances having an optimal heat loss function [96]. Heat stress causes a succession of dramatic alterations in rabbits’ biological functions, culminating in production impairment. By reducing heat-induced inhibition of osmotic equilibrium and preserving the tertiary structure of macromolecules in the kidney and other tissues, betaine, an osmolyte and a methyl group donor, may assist the animal in maintaining its thermoneutral state of homeostasis [97,98]. Pomegranate peel supplementation significantly increased the endogenous antioxidant status of rabbits, resulting in greater levels of SOD and TAC than in control rabbits. These enzymes, in conjunction with the exogenous low-molecular-weight antioxidant provided by whole-pomegranate extracts, contribute to the resistance against summer-induced oxidative stress by facilitating the removal of free radicals and other reactive species [99,100]. Despite the significant increases in SOD and TAC, current results indicate no significant differences in catalase activity between groups treated with vitamin C and organic selenium or between groups treated with organic selenium, betaine, and pomegranate peel. The dismutation of superoxide (O_2_) to H_2_O_2_ is the primary scavenging function of SOD, with catalase and/or GSH-Px functions serving as secondary to convert H_2_O_2_ to H_2_O [67,101]. Glutathione-peroxidase/glutathione control of H_2_O overflow appears to have been improved by the whole-pomegranate extract, but not catalase. Previous results have shown that polyphenols in pomegranate can interfere with the activity of genes and antioxidant enzymes and genes like catalase [99]. The antioxidant and antiapoptotic capacities of pomegranate phytochemicals are triggered under chronic summer heat stress, confirming the earlier claim that the antioxidant potential of pomegranate has a broad and less specific effect [99]. 

## 5. Conclusions

Although there was no negative group (not heat-stressed) in the experimental design, it can be concluded from the present study that dietary supplements of vitamin C (1000 mg vitamin C/kg diet), organic selenium (25 mg organic selenium/kg diet), betaine (1000 mg betaine/kg diet), and pomegranate peel (20 g pomegranate peel/kg diet) alleviated the negative effects of heat stress, which was reflected in the nutritional and physiological profile, in turn improving the performance, cecal fermentation and antioxidant status leading to the better health and productivity of rabbits. Further, among those examined supplements, betaine was found to be a superior one, due to the better nutritional and physiological profile as well as cecal fermentation of rabbits supplemented with betaine.

## Figures and Tables

**Table 1:** Ingredients and chemical composition of experimental diet.

Ingredients	(g/kg)	Chemical Analysis (g/kg as DM Basis)	
Alfalfa hay	313.0	Dry matter	901.7
Barley grain	247.0	Organic matter	939.6
Yellow corn	63.0	Crude protein	174.2
Wheat bran	101.0	Crude fiber	133.6
Soybean meal	219.0	Ether extract	31.7
Molasses	29.0	Nitrogen-free extract	600.1
Sodium chloride	5.0	Ash	60.9
Limestone	12.0	Neutral detergent fiber	344.8
Dicalcium phosphate	8.0	Acid detergent fiber	213.2
Minerals and vitamins mixture *	2.0	Acid detergent lignin	48.7
DL-methionine	1.0		

* Each kg contained: VA 6,000,000 IU; VD3 1,250,000 IU; VE 15,000 mg; VB1 1000 mg; VB6 1000 mg; VB12 6 mg; nicotinic acid 15,000 mg; pantothenic acid 5000 mg; biotin 50 mg; folic acid 500 mg; choline chloride 50% 400 mg; Mn 35 mg; Fe 40 mg; Cu 3.5 mg; Zn 25 mg; iodine 0.25 mg; Se 0.075 mg; cobalt 0.10 mg; CaCO_3_ 1000.

**Table 2:** Effect of vitamin C, organic selenium, betaine, and pomegranate peel supplementations on growth performance.

Items	Treatments					SEM	*p*-Value
	Control	Group A	Group B	Group C	Group D		
IBW, g	753.36	741.66	762.06	746.92	758.77	39.73	0.846
FBW, g	2359.82 ^c^	2651.72 ^b^	2754.66 ^b^	2923.71 ^a^	2669.32 ^b^	94.82	<0.001
ADG, g/d	22.95 ^c^	27.29 ^b^	28.47 ^b^	31.10 ^a^	27.29 ^b^	0.58	<0.001
FI, g/d	88.74	94.52	94.25	93.33	91.18	4.56	0.785
FCR	3.87 ^a^	3.46 ^b^	3.31 ^b^	3.00 ^c^	3.34 ^b^	0.14	0.018
Mortality, %	9.52 ^a^	4.76 ^b^	0.0 ^c^	0.0 ^c^	4.76 ^b^	0.11	<0.001

^a–c^ Means within a row with different superscripts are significantly different (*p* < 0.05). Group A: 1000 mg vitamin C/kg diet; group B: 25 mg organic selenium/kg diet; group C: 1000 mg betaine/kg diet; and group D: 20 g pomegranate peel/kg diet. IBW, initial body weight; FBW, final body weight; ADG, average daily gain; FI, feed intake; FCR, feed conversion ratio.

**Table 3:** Effect of vitamin C, organic selenium, betaine, and pomegranate peel supplementations on nutrient digestibility.

Items	Treatments					SEM	*p*-Value
	Control	Group A	Group B	Group C	Group D		
DM	59.52 ^c^	61.88 ^b^	62.16 ^b^	63.84 ^a^	62.31 ^b^	0.51	0.001
CP	58.59 ^d^	61.69 ^c^	63.08 ^b^	64.39 ^a^	63.22 ^b^	0.27	0.001
CF	43.28 ^c^	48.05 ^b^	48.61 ^b^	50.05 ^a^	48.83 ^b^	0.77	0.001
NFE	65.48 ^b^	68.67 ^a^	68.88 ^a^	69.51 ^a^	69.27 ^a^	0.89	0.009
NDF	57.55 ^c^	61.15 ^b^	61.71 ^ab^	62.36 ^a^	62.01 ^ab^	0.67	0.016
ADF	52.39 ^b^	55.91 ^a^	56.28 ^a^	56.69 ^a^	56.22 ^a^	0.81	0.022
DE (kcal/kg diet)	2447.73 ^c^	2487.87 ^b^	2529.88 ^a^	2542.92 ^a^	2521.77 ^a^	25.75	0.018

^a–d^ Means within a row with different superscripts are significantly different (*p* < 0.05). Group A, 1000 mg vitamin C/kg diet; group B, 25 mg organic selenium/kg diet; group C, 1000 mg betaine/kg diet; and group D, 20 g pomegranate peel/kg diet. DM, dry matter; CP, crude protein; CF, crude fiber; NFE, nitrogen-free extract; NDF, neutral detergent fiber; ADF, Acid detergent fiber; DE, Digestible energy.

**Table 4:** Effect of vitamin C, organic selenium, betaine, and pomegranate peel supplementations on carcass characteristics.

Items	Treatments					SEM	*p*-Value
	Control	Group A	Group B	Group C	Group D		
Carcass composition, g							
Live body weight	2129	2183	2245	2288	2206	159.92	0.846
Carcass weight	1165 ^c^	1297 ^b^	1336 ^b^	1428 ^a^	1311 ^b^	40.17	0.018
Dressing percentage	54.72 ^c^	59.41 ^b^	59.51 ^b^	62.41 ^a^	59.43 ^b^	0.21	0.001
Edible giblets, %							
Liver	5.52 ^a^	5.33 ^b^	5.29 ^b^	5.30 ^b^	5.31 ^b^	0.04	0.021
Kidney	1.44 ^a^	1.35 ^b^	1.33 ^b^	1.34 ^b^	1.33 ^b^	0.02	0.013
Heart	0.54	0.51	0.51	0.50	0.52	0.05	0.788
Carcass composition, %							
Moisture	73.17 ^a^	71.31 ^b^	71.18 ^b^	70.88 ^b^	71.22 ^b^	0.47	0.019
Protein	21.58 ^b^	22.38 ^a^	22.41 ^a^	22.69 ^a^	22.49 ^a^	0.33	0.011
Ether extract	4.79 ^a^	4.16 ^b^	4.09 ^b^	4.01 ^b^	3.89 ^b^	0.29	0.024
Total ash	1.37	1.34	1.41	1.44	1.39	0.17	0.792

^a–c^ Means within a row with different superscripts are significantly different (*p* < 0.05). Group A, 1000 mg vitamin C/kg diet; group B, 25 mg organic selenium/kg diet; group C,1000 mg betaine/kg diet; and group D, 20 g pomegranate peel/kg diet. Group A, vitamin C; group B, organic selenium; group C, betaine; and group D, pomegranate peel.

**Table 5:** Effect of vitamin C, organic selenium, betaine, and pomegranate peel supplementations on cecal fermentation and microbiota population.

Items	Treatments					SEM	*p*-Value
	Control	Group A	Group B	Group C	Group D		
Cecal fermentation patterns							
pH	6.36	6.32	6.38	6.29	6.31	0.18	0.793
NH_3_-N, mmol/L	12.48 ^a^	11.74 ^b^	11.65 ^b^	11.27 ^c^	11.42 ^bc^	0.14	0.012
Total VFA, mmol/L	63.22 ^b^	68.11 ^a^	68.18 ^a^	68.59 ^a^	68.41 ^a^	0.46	0.031
Acetic acid, mole, %	58.74 ^b^	60.82 ^a^	60.89 ^a^	61.33 ^a^	61.07 ^a^	0.58	0.006
Propionic acid, mole, %	19.57 ^b^	22.21 ^a^	22.26 ^a^	22.69 ^a^	22.41 ^a^	0.47	0.002
Butyric acid, mole, %	8.64	8.49	8.44	8.31	8.37	0.39	0.683
Cecal microbial count (log cfu/g cecal digesta)							
Total bacterial count	4.85 ^b^	5.77 ^a^	5.85 ^a^	6.26 ^a^	6.11 ^a^	0.47	0.021
*Enterococcus*	3.17 ^a^	1.25 ^b^	1.19 ^b^	1.15 ^b^	1.03 ^b^	0.25	0.017
Total coliforms	2.78 ^a^	1.13 ^b^	1.11 ^b^	1.07 ^b^	1.01 ^b^	0.11	0.011
*E. coli*	3.42 ^a^	1.29 ^b^	1.25 ^b^	1.17 ^b^	1.15 ^b^	0.14	0.022

^a–c^ Means within a row with different superscripts are significantly different (*p* < 0.05). Group A, 1000 mg vitamin C/kg diet; group B, 25 mg organic selenium/kg diet; group C, 1000 mg betaine/kg diet; and group D, 20 g pomegranate peel/kg diet.

**Table 6:** Effect of vitamin C, organic selenium, betaine, and pomegranate peel supplementations on serum biochemistry and antioxidants indices.

Items	Treatments					SEM	*p*-Value
	Control	Group A	Group B	Group C	Group D		
Total protein, g/dL	6.47 ^c^	6.85 ^b^	6.94 ^b^	7.22 ^a^	6.97 ^b^	0.13	0.001
Albumin, g/dL	3.66 ^c^	3.84 ^b^	3.91 ^b^	4.11 ^a^	3.92 ^b^	0.07	0.001
Globulin, g/dL	2.81 ^b^	3.01 ^a^	3.03 ^a^	3.11 ^a^	3.05 ^a^	0.10	0.017
TotalCholesterol, mg/dl	93.55 ^a^	86.28 ^b^	85.77 ^b^	83.28 ^c^	81.66 ^c^	2.51	0.029
Triglycerides, mg/dL	66.73 ^a^	62.59 ^b^	62.11 ^b^	61.06 ^b^	60.35 ^b^	2.37	0.015
Creatinine, mg/dL	0.93 ^a^	0.89 ^b^	0.88 ^b^	0.81 ^c^	0.81 ^c^	0.02	0.001
Urea, mg/dL	42.84 ^a^	40.18 ^b^	40.27 ^b^	39.44 ^c^	38.83 ^c^	1.55	0.016
Antioxidant status							
SOD, U/mL	7.57 ^c^	8.62 ^b^	8.85 ^b^	9.55 ^a^	9.71 ^a^	0.35	0.001
CAT, U/mL	9.33 ^c^	9.94 ^b^	10.31 ^ab^	10.74 ^a^	10.89 ^a^	0.41	0.027
GSH, U/mL	1.44 ^c^	2.67 ^b^	2.88 ^b^	3.03 ^a^	3.22 ^a^	0.39	0.009
GPx, U/mL	6.37 ^c^	6.98 ^b^	7.05 ^b^	7.26 ^a^	7.44 ^a^	0.25	0.011
MDA, U/mL	1.37 ^a^	0.89 ^b^	0.88 ^b^	0.76 ^b^	0.71 ^b^	0.19	0.022

^a–c^ Means within a row with different superscripts are significantly different (*p* < 0.05). Group A, 1000 mg vitamin C/kg diet; group B, 25 mg organic selenium/kg diet; group C, 1000 mg betaine/kg diet; and group D, 20 g pomegranate peel/kg diet. SOD, superoxide dismutase; CAT, catalase; GSH, reduced glutathione; GPx, glutathione peroxidase; MDA, malondialdehyde.

## Data Availability

Data are contained within the article.
